# Missense mutation of *VKORC1* leads to medial arterial calcification in rats

**DOI:** 10.1038/s41598-018-31788-6

**Published:** 2018-09-13

**Authors:** Arnaud Michaux, Benjamin Matagrin, Jean-Valéry Debaux, Leon J. Schurgers, Etienne Benoit, Virginie Lattard

**Affiliations:** 1 USC 1233 RS2GP, INRA, VetAgro Sup, Univ Lyon, F-69280 Marcy l’Etoile, France; 2 Department of Biochemistry, Cardiovascular Research Institute Maastricht (CARIM), Maastricht University, Maastricht, The Netherlands

## Abstract

Vitamin K plays a crucial role in the regulation of vascular calcifications by allowing activation of matrix Gla protein. The dietary requirement for vitamin K is low because of an efficient recycling of vitamin K by vitamin K epoxide reductase (VKORC1). However, decreased VKORC1 activity may result in vascular calcification. More than 30 coding mutations of *VKORC1* have been described. While these mutations have been suspected of causing anticoagulant resistance, their association with an increase in the risk of vascular calcification has never been considered. We thus investigated functional cardiovascular characteristics in a rat model mutated in *VKORC1*. This study revealed that limited intake in vitamin K in mutated rat induced massive calcified areas in the media of arteries of lung, aortic arch, kidneys and testis. Development of calcifications could be inhibited by vitamin K supplementation. In calcified areas, inactive Matrix Gla protein expression increased, while corresponding mRNA expression was not modified. Mutation in *VKORC1* associated with a limited vitamin K intake is thus a major risk for cardiovascular disease. Our model is the first non-invasive rat model that shows spontaneous medial calcifications and would be useful for studying physiological function of vitamin K.

## Introduction

Vascular calcification is a pathology related to age which affects most people over 60 years. It is characterized by deposits of hydroxyapatite crystals in the media and/or intima of the arterial walls. This calcification can lead to complications like high blood pressure or heart failure which are major risk factors for cardiovascular disease. Calcification is accelerated by inflammation, diabetes, chronic kidney disease (CKD), osteoporosis or vitamin K antagonist treatment like warfarin^1^. It is widely accepted that vascular calcification is not the consequence of a passive phenomenon but is regulated by complex active mechanisms^1^, especially vitamin K-dependent mechanisms. It has been demonstrated that vitamin K plays a crucial role in the regulation of ectopic calcifications by either holding progression or even reversing vascular calcification^2^. Vitamin K is an essential co-factor for the gamma-glutamyl-carboxylase enzyme, which is responsible for activation of 17 vitamin K-dependent proteins (VKDP) by gamma-carboxylation of their glutamate residues. Carboxylation confers these so-called vitamin K-dependent proteins (VKDP) the ability to bind calcium. Among these proteins, factors II, VII, IX and X are involved in the coagulation process. Extrahepatic VKDP such as matrix-Gla-protein (MGP), osteocalcin or Gla-rich protein^3^ are involved in bone and calcium metabolism. MGP, when carboxylated, is a potent inhibitor of vascular calcification and knockout of the MGP gene in mice results in premature death by spontaneous calcification of arteries^4^.

Despite the conversion of vitamin K in vitamin K epoxide by the gamma-glutamyl-carboxylase to activate VKDP, the daily requirements for vitamin K in normal adult are low with recommendations for the different countries comprised between 50 to 120 µg/day/adult^5^. The low requirement can be explained by an efficient recycling system of vitamin K from vitamin K epoxide by the vitamin K epoxide reductase enzyme (VKORC1) allowing the use of vitamin K for some 500 carboxylation reactions^6^.

Failure in the reduction of vitamin K epoxide by VKORC1 may result in loss of inhibition of vascular calcification and thus may cause accelerated vascular calcification. Reduction in VKORC1 activity results from the daily use of anticoagulant therapy with vitamin K-antagonists to prevent and treat thromboembolic disorders^7^. It is now accepted that the long-term use of anticoagulant induces vascular calcification^8–14^. Additionally, experimental studies in rats and mice have clearly shown that inhibition of VKORC1 by warfarin induces medial calcification of the arterial vessel wall^15–17^.

This reduction in VKORC1 activity may also result from sequence variations of the *VKORC1* gene. 5 common non-coding polymorphisms of the *VKORC1* gene have been identified in patients requiring lower doses of warfarin, including polymorphisms in the promoter region, in the first and second intron, and in the 3′ downstream region^18–20^. Since the presence of these polymorphisms is associated with reduced expression of VKORC1^21^, clinical studies have attempted to link these polymorphisms to an increased risk of vascular calcification. The results thus far are conflicting and do not allow a conclusion^20,22–26^. More than 30 mutations in the coding region of VKORC1 have also been described in patients requiring increased doses of anticoagulants^21^. While these mutations have been widely suspected of causing anticoagulant resistance, their association with an increase or decrease in the risk of vascular calcification has never been considered. However, some of these mutations, such as mutations at amino acid 139^27^, are localized in the substrate binding site and may induce a decrease in activity of VKORC1. The objective of this study is to study the impact of such mutation in the development of vascular calcifications by using a rat model carrying a mutation of *VKORC1* at position 139.

## Results

### The genotype of F10-introgressed rats did not result in phenotype modification

From 41 crosses with heterozygous F10-introgressed rats, 35 litters were obtained comprising the 3 different genotypes YY, YC, CC with a distribution of Mendelian type in terms of sex and genotype. At the age of 2 months, 11 males and females of the same genotype were mated. The average duration between presentation of the male to the female and the birth of the pups was 24.7 (±1.01), 25.5 (±1.04) and 24.46 (±0.93) days respectively for the crossing YY*YY, YC*YC and CC*CC. The litter size was 11.9 (±2.39), 13.1 (±2.21) and 12.6 (±2.88) for the YY*YY, YC*YC and CC*CC crossings. The morphological aspect and the weight of the pups from the different crosses were not differentiable at the time of weaning at the age of 30 days. To eventually detect phenotypic variations between genotypes, 9 male rats of each genotype fed with a standard diet were observed for 12 months. During this period, 1 single YC genotype rat was found dead at the age of 9 months. Necropsy analysis of this rat did not reveal any sign of hemorrhage. None of the other rats exhibited bleeding or increased prothrombin time during this period. The weight gain was not significantly different between genotypes throughout the duration of the diet with a 12-month weight of 480 (±88.4), 511 (±104.6), 520 (±67.6) grams for YY, YC and CC groups respectively (Suppl. information). Plasma urea, creatinine, calcium and phosphate concentration were not affected by the genotype and mean values are reported in Table 1.

**Table 1:** Weight and biochemical parameters of homozygous YY, CC and heterozygous YC rats aged of 16 weeks after a vitamin K_3_ deficient diet (−K3) or standard diet (+K3) feeding for 12 weeks.

Rats	Weight^(g)^	Creatininemia (µM)	Uremia (mM)	Phosphatemia (mM)	Calcemia (mM)	[MK4] in testis (% compared to YY^+K3^)
YY^+K3^	335 ± 19.3	22.0 ± 1.9	335 ± 19.3	1.90 ± 0.17	2.64 ± 0.19	100 ± 9.4
CC^+K3^	368 ± 58.0	24.4 ± 4.25	368 ± 58.0	1.89 ± 0.22	2.62 ± 0.08	52 ± 5.7
YY^−K3^	357 ± 21.6	24.3 ± 4.25	357 ± 21.6	1.72 ± 0.21	2.60 ± 0.12	61.3 ± 3.9
CC^−K3^	320 ± 49.8	22.2 ± 1.48	320 ± 49.8	1.74 ± 0.25	2.59 ± 0.09	13.1 ± 1.7

Results expressed as mean ± SD. n = 6 for groups for YY^+k3^ and YY^−K3^ groups and n = 11 and 10 for groups CC^+K3^ and CC^−K3^ respectively.

### Feeding of F10-introgressed rats for 12 weeks with a special diet-K3 did not result in morphological, coagulation or biochemical plasma modifications whatever the genotype was

**Figure 1:**
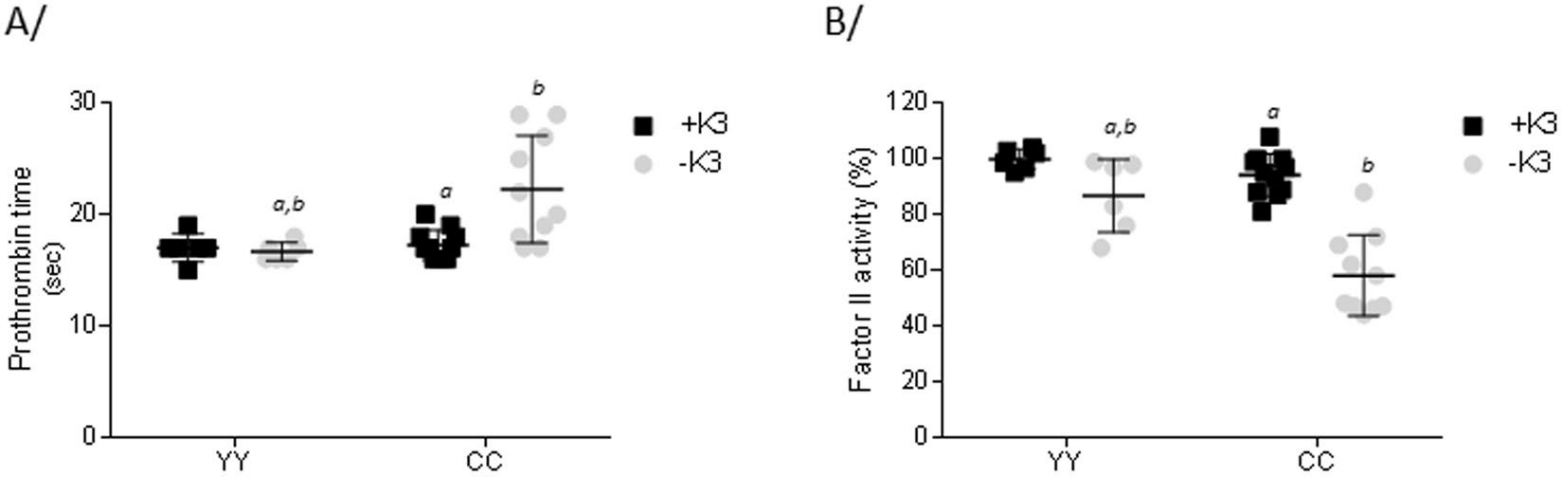
Influence of the diet on the coagulation function in YY and CC rats **A**/Prothrombin time expressed in seconds and **B**/factor II activity expressed as a percentage compared to the mean value obtained for YY^+K3^ group. In black squares, rats fed with standard diet; in grey circles, rats fed with diet –K3, during 12 weeks after the weaning. Results expressed as mean ± SD. n = 6 for groups YY and n = 11 and 10 for groups CC^+K3^ and CC^−K3^ respectively. *p < 0.05 vs YY^+K3^ group. Statistical analysis was performed using Tukey’s multiple comparisons test. Statistic difference was reported when only a unique variable between groups is modified (either genotype or diet). *P* < 0.01 was the accepted level of significance. *a,b,c*: statistical difference between 2 groups.

After receiving 12 weeks of special diet −K3 *ad libitium* just after the weaning, the average weight of YY or CC rats was 357 ± 22 grams and 320 ± 50 grams and weights were not statistically different between genotypes (Table 1). The vitamin K deficiency led to the death of two animals CC^−K3^ out of twelve. Necropsy analysis of these rats revealed the presence of large blood clots localized at the chest and abdomen. The mean prothrombin time of CC^−K3^ group was only increased by 5.3 seconds compared to group YY^+K3^ (Fig. 1A). Along with this slight increase in prothrombin time, activity of clotting factor II was reduced by 41.9% in the group CC^−K3^ *vs* YY^+K3^ (Fig. 1B). No uremia, creatininemia, calcemia and phosphatemia was induced by the feeding with the special diet –K3 (Table 1).

### CC rats receiving a specific diet –K3 for 12 weeks present vitamin K deficiency and tissue-calcifications

To evaluate the vitamin K status in YY^+K3^, YY^−K3^, CC^+K3^ and CC^−K3^ rats, menaquinone-4 concentrations were determined in testis Results are presented in Table 1. In testis of CC rats, menaquinone-4 concentrations were shown to be reduced by 50 and 90% in CC^+K3^ and CC^−K3^ rats, respectively, compared to YY^+K3^.

**Figure 2:**
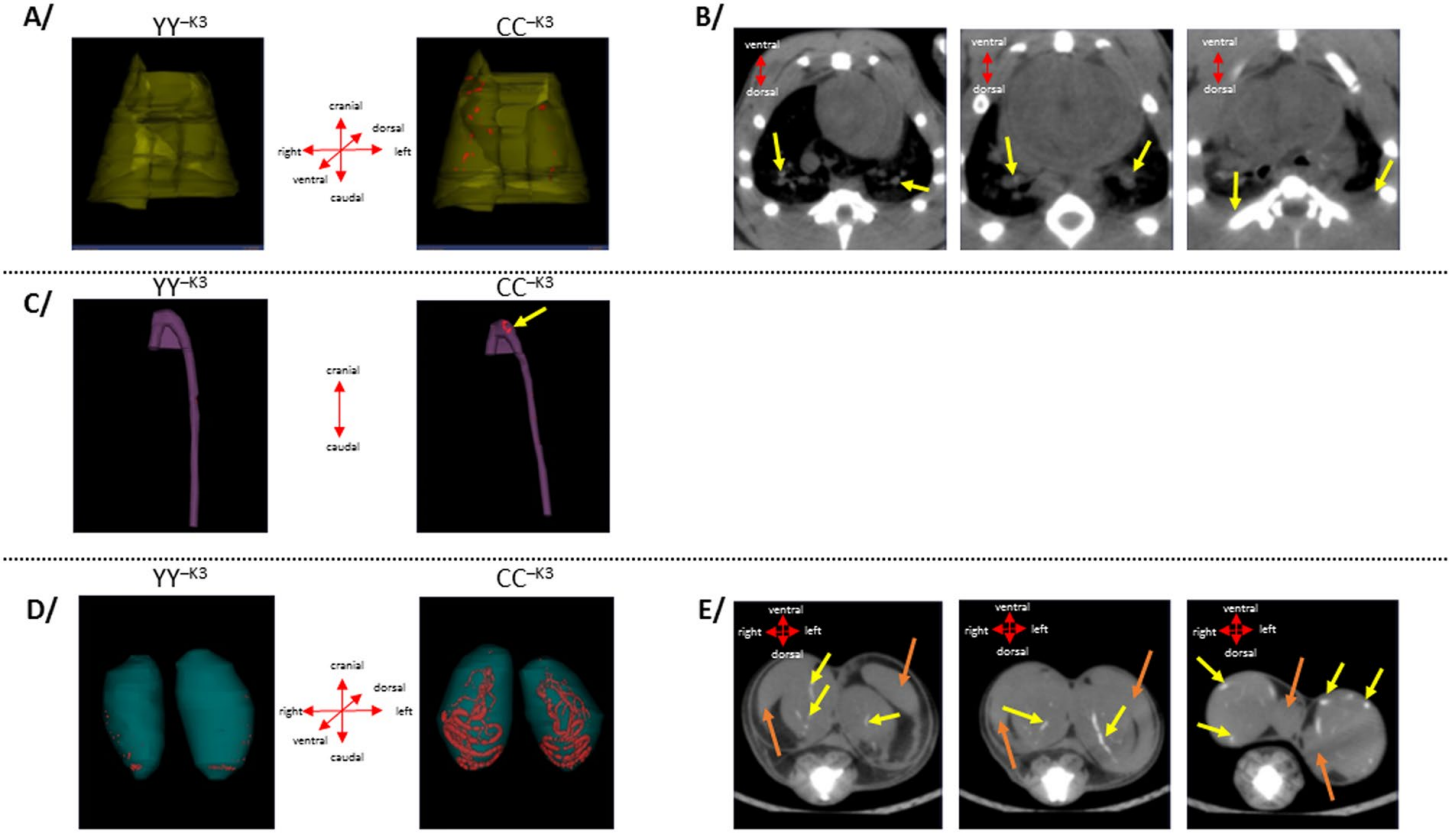
µCT analysis of various tissues of YY^−K3^ and CC^−K3^ rats. In **A**/3D-representations of calcification volume (in red) inside lung (in yellow), in **B**/axial μ-CT views of calcifications present at the lung vessels of CC^−K3^ rats (calcifications are indicated by yellow arrows), in **C**/3D-representations of calcification volume (in red) inside aortic arch (in purple), in **D**/3D-representations of calcification volume (in red) inside testis (in grey), in E/axial μ-CT views of calcifications present in testis of CC^−K3^ rats (calcifications are indicated by yellow arrows and epididymis is indicated by orange arrows).

To take an overall view, development of extraosseous calcification was first analyzed by µCT in the four target zones (lung, aortic arch, kidneys and testis) from one individual from each group of rats (i.e., YY or CC rats) which had received either a normal diet or a specific diet –K3. Calcification zones were detected in the lung, aortic arch and testis of the CC^−K3^ rat whereas the other rats YY^+K3^, YY^−K3^ and CC^+K3^ did not show calcification in these regions (Fig. 2). High–vitamin K intake (K_1_ or K_3_) given in CC rats all along the feeding period with diet-K3 weeks prevented the observation of calcification zones in lung, aortic arch and testis.

After acquisition of images by µCT scanning, 3D-representations were made to represent calcifications within target regions in CC^−K3^ rat and calculate the global volume occupied by these calcifications. In lungs, the areas of calcification were mainly observed in the pulmonary vessels, as shown by the µCT axial view (Fig. 2B). The three-dimensional representation of the lung enables to estimate the volume of these calcifications at ~3.4 mm^3^. In the aortic arch, the volume of calcifications was estimated at 1.2 mm^3^. The most significant areas of calcification were observed in the right and left testis of the CC^−K3^ rat, with volumes of 63.2 mm^3^ and 60.5 mm^3^, respectively. These calcifications appeared to be inside the testicular blood vessels. The small volumes detected in testis of rats belonging to the other groups were only due to the presence of artifacts related to the presence of tail bones near the target region. In kidney, no calcification was visualized by μCT whatever the group of rats considered.

**Figure 3:**
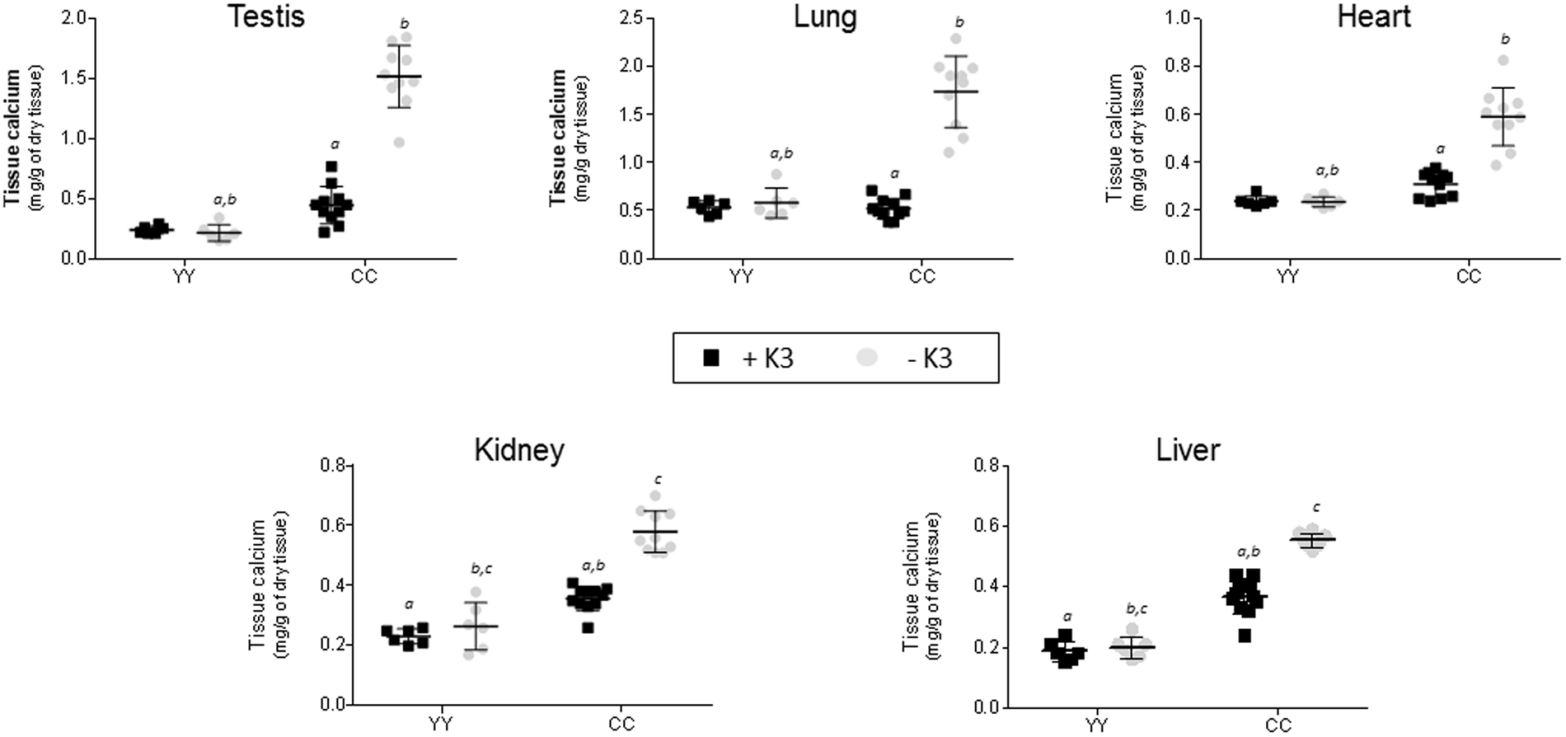
Tissue calcium content in testis, lung, heart, kidney and liver of 4 months old-YY or CC rats fed with standard diet (black square) or special diet –K3 (gray circle) since the weaning. Results are expressed as mean ± SD. n = 6 for groups YY and n = 11 and 10 for groups CC^+K3^ and CC^−K3^ respectively. Statistical analysis was performed using Tukey’s multiple comparisons test. Statistic difference was reported when only a unique variable between groups is modified (either genotype or diet). *P* < 0.01 was the accepted level of significance. *a,b,c*: statistical difference between 2 groups.

To precisely compare calcification process according to the genotype, age and diet, tissue calcium concentration was determined in YY, YC and CC rats. No modification of calcium contents in liver, heart, lung, kidney, aorta and testis was detected between genotypes in one year-old rats fed with standard diet for 12 months (Table 2). On the contrary, rats fed with special diet –K3 for 12 weeks after the weaning, difference in calcium contents were detected between genotype in 4 months-old rats. In the CC^−K3^ group, calcium concentration was significantly increased in all tissues observed compared to the 3 other groups (Fig. 3). This increase was 2.8 times higher in liver, 2.5 times in heart, 3 times in lung and 7.1 times in testis compared to the YY^+K3^ group. A 51% increase in calcium concentration in the liver was also observed in the group CC^−K3^ compared to the group CC^+K3^.

**Table 2:** Tissue calcium concentration of 1 year old-YY, YC and CC rats fed with standard diet.

Rats	Tissue calcium (µg/g of dry tissue)					
	In liver	In heart	In kidney	In lung	In testis	In aorta
YY	244 ± 31.7	331 ± 42.8	510 ± 133	786 ± 228	341 ± 66.8	429 ± 111
YC	231 ± 36.0	325 ± 30.1	459 ± 136	788 ± 195	352 ± 52.5	478 ± 130
CC	218 ± 46.4	308 ± 31.4	537 ± 136	857 ± 267	343 ± 38.1	478 ± 205

Results are expressed as mean ± SD. n = 9 for YY and CC groups, n = 8 for YC group.

### Tissue-calcium deposits are located in the medial layer of arteries

**Figure 4:**
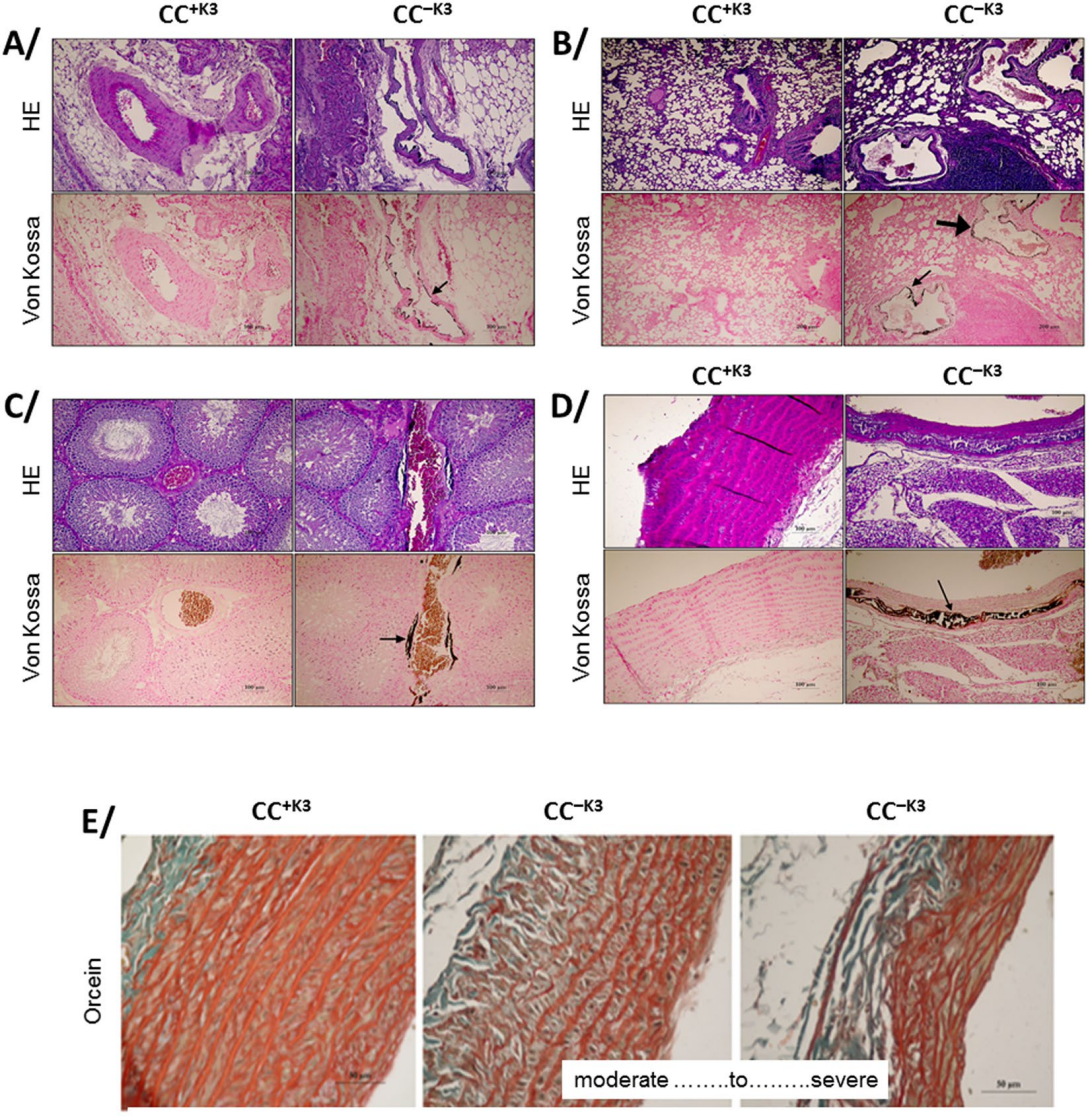
Von-Kossa, hematoxylin-eosin (HE) and orcein staining of various tissues of CC rats feed with (CC^+K3^) or without (CC^−K3^) vitamin K_3_ during 12 weeks. **A/**kidney, **B/**lung, **C/**testis, **D** and **E/**aortic cross. Black arrow points out calcifications.

Von Kossa staining did not allow to detect calcium deposit in the different organs (i.e., kidney, lung, testis and aortic arch) obtained from YY^+K3^, YY^+K3^, CC^+K3^ (Fig. 4). On the contrary, feeding for 12 weeks with vitamin K_3_ deficient diet of CC rats resulted in significant calcifications in lung and testis (Fig. 4). Theses calcifications were present in 100% of the samples and were localized in the media of lung and testicular arteries. Some calcifications were also observed in the media of arteries located in the renal hilum (6 samples positives on 10) (Fig. 4) and in the media of the aortic cross (3 samples positives on 10) (Fig. 4) in these animals. A moderate to severe destructuration in the proximal aorta of CC^−K3^ rats with alteration of elastin fibers was visible in orcein staining, while no alteration of the elastin fibers was found with a standard regime in the CC^+K3^ group (Fig. 4E). No calcium deposit was detected in the liver of CC^−K3^ rats.

### Calcification staining is positively correlated with immunohistochemical ucMGP staining

To investigate matrix Gla protein in relation to calcification of vascular tissue, MGP mRNA level was quantified in testis, lung and kidney according to the genotype and diet. mRNA expression of MGP was not significantly different in CC-K3 compared to other groups in any of the tissues analysed (data not shown).

**Figure 5:**
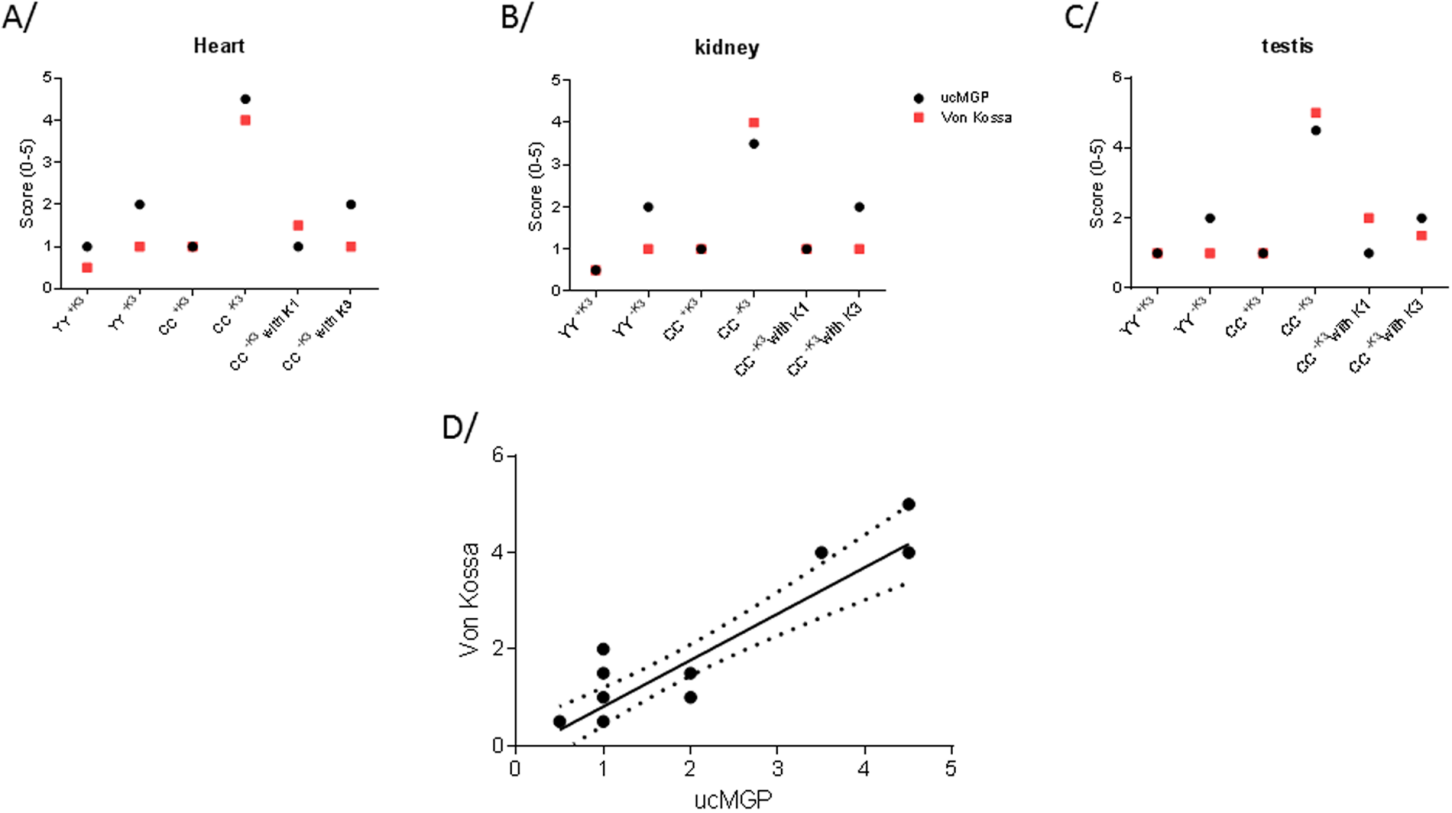
Score of ucMGP immunostaining and Von Kossa staining in heart (**A**), kidney (**B**) and testis (**C**) and correlation between ucMGP and Von Kossa staining (**D**). In D, the line corresponds to the linear regression with its 95% confidence interval in dot line.

Subsequent staining with conformation-specific antibodies against ucMGP (Suppl. information) revealed high staining in heart, kidney and testis of CC rats fed with diet −K3, whereas low staining was detected in other groups (Fig. 5). Moreover, addition of vitamin K1 or vitamin K3 during the feeding period with diet –K3 prevented increased ucMGP presence. These immunohistochemical stainings enabled to establish a positive correlation between Von kossa staining and ucMGP staining (Fig. 5D), suggesting that a limited intake of vitamin K in CC rats led to calcification phenotype by inhibiting carboxylation of MGP.

## Discussion

The results presented herein clearly demonstrate the development of extensive medial arterial calcifications in CC rats when these rats are fed with a diet −K3. To induce this phenotype, the association of two determining factors is strictly required: 1/a limited vitamin K intake in the diet, and 2/the presence of the p.Y139C mutation in the *VKORC1* gene. Indeed, CC rats fed with a standard diet did not develop any arterial calcification throughout the observation period while the same rats fed with diet −K3 developed extensive arterial calcifications. Of note, the diet −K3 used in this study differs from the standard diet by the absence of supplementation in vitamin K3, but also by a different composition. The barley rich in vitamin K1 was replaced by corn starch. In order to prove the dependence of the observed phenotype on the vitamin K intake, daily vitamin K3 or K1 supplementation was performed in CC rats fed with diet −K3. No arterial calcification could be detected in these animals, which proves the unequivocal role of vitamin K in vascular calcification.

The presence of the p.Y139C mutation in the *VKORC1* gene is also a fundamental factor in the development of these arterial calcifications. In YY rats, a limited intake of vitamin K did not induce development of medial arterial calcifications or an increase in tissue calcium concentrations. Consequently, in these rats, despite a limited dietary intake of vitamin K, the efficient recycling of vitamin K by VKORC1 enzyme together with the production of vitamin K by the microflora ensures the vitamin K requirements that are necessary for activation of VKDPs involved in the inhibition of tissue calcification. This balance seems to be completely disturbed in CC rats. Indeed, in these rats, a limited dietary intake of vitamin K results in the development of massive and disseminated arterial calcifications. The presence of aortic and renal ectopic vascular calcifications in wild rats homozygous for Y139C mutation trapped in the field had been previously described by Kohn *et al*.^28^. Nevertheless, because these rats were wild rats trapped in the field, it was difficult to draw conclusions since the genetic background could not be controlled. These rats were at least carriers for the p.Y139C mutation in the *VKORC1* gene, but these rats could also carry other point mutations in the rest of their genome. In addition, because these rats were wild rats, their microflora could be altered and the development of calcifications could be due to this alteration. In fact, sterilization of microflora in laboratory rats or mice receiving a limited intake of vitamin K has been shown to induce the development of arterial calcifications^29,30^. The Y139C mutation into a laboratory rat genome according to the introgression protocol performed in this study enables to demonstrate without ambiguity the involvement of the Y139C mutation in the observed phenotype. Indeed, even if a wild rat carrying the Y139C mutation was initially used to generate the introgressed rat strain, the successive introgression over 10 generations of the Y139C mutation in Sprague Dawley rats allows us to exclude the involvement of other point mutations. Such an introgression protocol had already been applied to evaluate the consequences of another mutation of *VKORC1*, the Y139F mutation. After 9 generations (i.e., one less than for this introgression), the size of the introgressed chromosome fragment in the laboratory rat genome was estimated to be less than 28 Mb (i.e., less than 1 tenth of the whole chromosome 1)^31^. In this experiment, because we used one supplementary generation, the size of the introgressed fragment is probably even lower. A different composition of the intestinal microflora between the genotypes also seems totally excluded because of the protocol. Indeed, after 10 generations of backcrossing between heterozygous rats from the previous generation and Sprague Dawley rats, an intercross between F10 heterozygous males and females was performed to generate the different genotypes (i.e., YY rats and CC rats). The composition of their intestinal microflora between genotypes is thus undoubtedly identical and cannot be responsible for the difference in phenotype. The phenotype observed (i.e., the sensitivity of the arterial media to calcification induced by a limited intake of vitamin K) is thus associated to the Y139C genotype.

Mutations in the *VKORC1* gene are usually considered as linked to resistance to anticoagulants either in humans^32^ or in rats^33^. Y139C mutation results in a strong resistant to anticoagulant such as warfarin. Nevertheless, this mutation also induces a decrease in the efficiency of the VKORC1 enzyme to recycle vitamin K. It has been shown that the catalytic efficiency of the VKORC1 Y139C protein is reduced by a factor of 20^34^. This altered recycling of vitamin K is further aggravated by the fact that the Y139C mutation leads to change in the catalysis of vitamin K epoxide by VKORC1 and one third of the vitamin K epoxide is converted into 3-hydroxyvitamin by the mutated VKORC1 instead of being recycled to vitamin K^34^. Loss of vitamin K as 3-hydroxyvitamin K associated with inefficient recycling is undoubtedly responsible for additional vitamin K requirements that cannot be covered by intestinal microflora production. These increased vitamin K requirements in rats with mutations in the *VKORC1* gene have been suggested by earlier studies^35,36^ and results obtained in this study confirm these observations.

In humans, nearly 30 coding mutations of *VKORC1* have been detected. Among these almost 30 mutations, 16 result in a very strong *in vitro* decrease of the efficiency of VKORC1 to recycle vitamin K^32^ even though characterizations of catalytic properties of mutants have been sometimes conflicting^37,38^. Among these mutations, only one was shown to be associated to hemostatic defect, the R98W found in Libanese family and people with this specific mutation required vitamin K1 supplementation to survive^39^. The other mutations have been discovered in patients requiring increased doses of vitamin K antagonists only when these patients required thromboembolic treatment^27^. Nevertheless, most of these mutations have been reported to be in the heterozygous state. It is thus probable that some of these mutations are associated with higher vitamin K requirements. However, the frequency of these mutations has never been considered in people with medial arterial calcification and studies to reveal such association should be considered. Moreover, it has been described that CKD is often associated with a vitamin K deficiency. This deficiency is only partially understood. Reduced vitamin K intake^40^ and reduction of VKORC1 expression^16^ have been suggested. The frequency of mutations of the *VKORC1* gene in CKD patients with deficiency in vitamin K could also be considered as risk factor for vitamin K deficiency.

After 3 months of diet −K3, all the CC rats present medial arterial calcifications. Clear calcifications are detected in lung and aortic cross by µCT. Such calcifications have been already observed in rats receiving high dose of warfarin^15^. Surprisingly, severe and extensive calcifications are also observed by µCT in testis of all CC rats receiving limited intake of vitamin K. In this study, testis seems a tissue particularly sensitive to medial arterial calcification induced by vitamin K deficiency. Vitamin K concentrations have been reported to be particularly high in testis of rats maintained under normal feeding conditions compared to other tissues^41^, which is consistent with the high sensitivity of this tissue to vitamin K deficiency shown herein. This susceptibility had never been reported until now, even in the warfarin-mediated calcification model^15^. It is possible that the strong expression of VKORC1L1 in testis^42^, a paralogue enzyme of VKORC1 able to recycle vitamin K as VKORC1, but unlike VKORC1 highly resistant to warfarin^42^, preventing the development of vitamin K-dependent medial arterial calcification when warfarin is used. The other tissues considered in this study appear less sensitive to medial arterial calcification and detection techniques more sensitive than μCT are necessary to highlight them.

**Figure 6:**
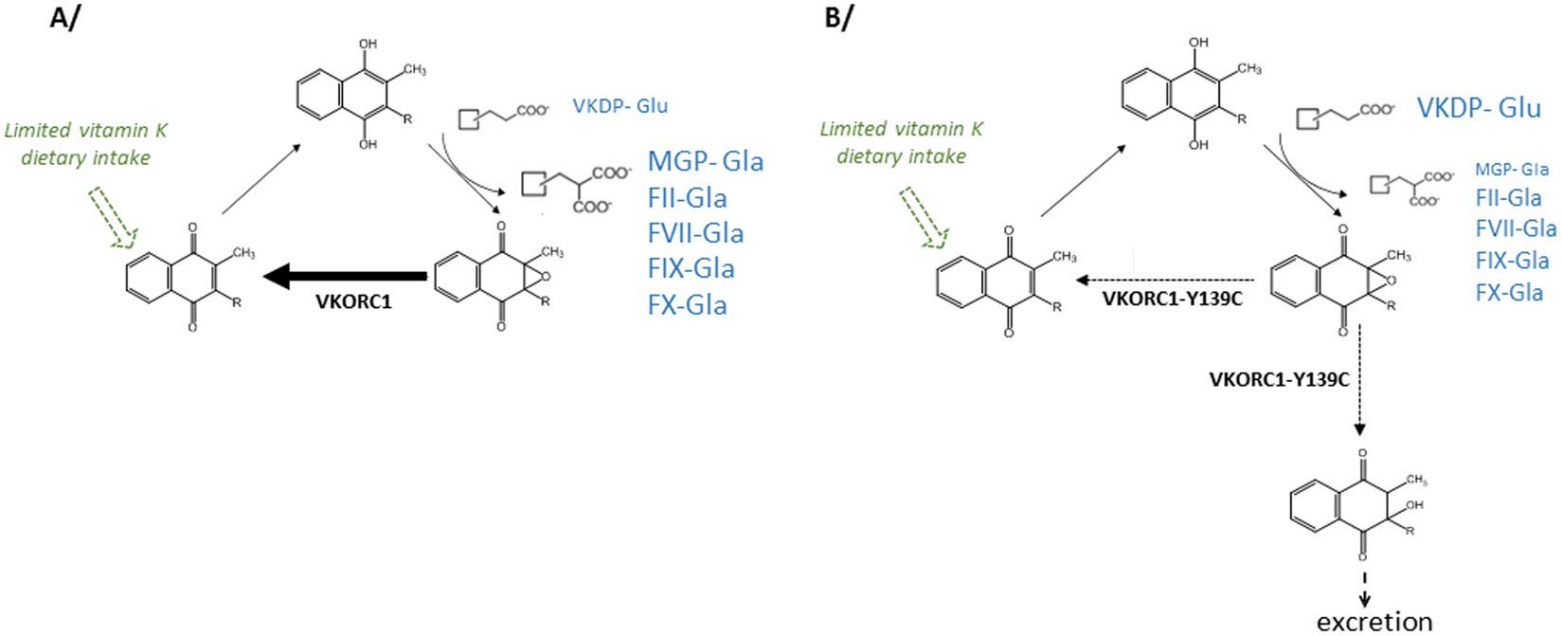
Mechanism of the development of vascular calcification in CC rat fed with a normal diet not supplemented with vitamin K3.

These media arterial calcifications are most likely due to the absence of gamma-carboxylation of MGP. Indeed, a positive correlation was systematically found between accumulation of calcium and accumulation of undercarboxylated MGP. The crucial function of MGP in the calcification process^43^ is accepted and absence of activation of MGP due to vitamin K deficiency can easily explain the development of medial arterial calcification in CC rats (Fig. 6). Surprisingly, while MGP is not gamma-carboxylated in CC rats fed with a diet −K3, activation of clotting factors by gamma-carboxylation seems to be poorly impacted.

Animal models of vitamin K deficiency to study medial arterial calcifications are lacking. Indeed, experimental vitamin K deficiency can be obtained using synthetic diets. In this case, absence of vitamin K dietary intake can cause drastic decrease in activated clotting factors and leads to hemorrhagic syndrome^44,45^. In our model, the limited intake of vitamin K associated with the presence of the VKORC1 mutation leads to decrease of the pool of activated vitamin K-dependent clotting factors but not sufficiently to lead to hemorrhagic syndrome. Indeed, to modify the prothrombin time in rats, it was demonstrated that reduction of at least 70% of activity of factor II was required^46^. This reduction in our model is only of 40%. This limited reduction can be explained by the triage theory proposed by McCann and Ames^47^ with a strong first-pass effect of the liver on dietary vitamin K. Rats treated with warfarin (100 mg/kg/day)^15^ present arterial calcifications only in growing animals but less in older, non-growing animals. Moreover, the dosage necessary to induce calcification appears to be pharmacological high and implies the use of large amount of vitamin K1 to avoid a lethal hemorrhagic syndrome. A recent rat model using adenine in order to create uremia appears to be interesting because the use of vitamin K1 is not necessary^48^, but this model does not allow to observe microscopy detectable calcifications. CC rats might be an interesting model to study vitamin K deficiency in relation to medial arterial calcifications. When CC rats are maintained with standard diet with vitamin K3, they do not present any calcification, reproduce and grow normally, and have no coagulation disorders and no biochemical changes. Implementation of this model does not involve any invasive action, it is based only on giving to CC rats a normal diet not supplemented with vitamin K3. In this way, mortality by hemorrhage is extremely rare and development of medial arterial calcifications is reproducible and systematic. In conclusion, our model is the first non-invasive rat model that shows spontaneous medial calcifications. Our model could also be crucial for studying the physiological importance of vitamin K.

## Methods

### Animals

Wild rats *Vkorc1*^*C139/C139*^ strain, carrying the Y139C mutation in *Vkorc1* at the homozygous state, was a generous gift from Julius Kuhn Institute. The founder animals of this strain were initially trapped in Germany. F10-introgressed rats homozygous for C139, F10-introgressed rats homozygous for Y139 or F10-introgressed rats heterozygous were derived from backcrossings and intercrossings between Sprague-Dawley rats (designed as recipient strain) (Charles River, St Germain sur l′Arbresles, France) and rats from wild rats VKORC1^*C139/C139*^ strain (designed as donor strain for the Y139C mutation) (Suppl. information). To form congenic strains, four male wild rats VKORC1^*C139/C139*^ were crossed with four Sprague-Dawley females to create the F1 hybrid generation, respectively. The F1 heterozygous for the Y139C mutation males were backcrossed to Sprague-Dawley females to give the F2 generation. The genotype of F2 young rats was determined by the allele-specific PCR method, as described below. Heterozygous females were backcrossed to the recipient Sprague-Dawley strain for 8 additional generations yielding F10 generation. Finally, an F10 intercross of males heterozygous for Y139C with females heterozygous for Y139C was carried out to obtain F10-introgressed rats homozygous for Y139 (designed in this study as YY), F10-introgressed rats homozygous for C139 (designed in this study as CC), and F10-introgressed rats heterozygous for Y139C (designed in this study as YC).

Animals were kept in standard cages (Eurostandard, Type IV, Tecniplast, Limonest, France), with a day-night cycle 12 h/12 h and received water and in standard condition, standard feed containing vitamin K_3_ (Scientific Animal Food and Engineering, reference A04) (designed in this study as diet +K3), both *ad libitum*.

### Genetic characterization of animals

Genomic DNA was extracted from tail using the Extracta™ DNA Prep for PCR – Tissue extraction kit (Quanta, Biosciences). The allele-specific PCR method employed two pairs of primers purchased from Eurogentec (Angers, France). The forward primer was one of the two primers which the three prime nucleotide of the first one matched the *Vkorc1* sequence of the SD strain (i.e. the rat genome sequence of *Vkorc1*; VKORC1^*Y139/Y139*^-primer: 5′-CATTGTTTGCATCACCACCTA-3′) and the three prime nucleotide of the other matched the mutation in the *Vkorc1* sequence of the donor strain (VKORC1^*C139/C139*^-primer: 5′-CATTGTTTGCATCACCACCTG-3′). The reverse primers matched the Vkorc1 sequence of the rat genome sequence for the *Vkorc1* (5′-TCAGGGCTTTTTGACCTTGTG-3′). We amplified 135 base pairs of *Vkorc1* gene using quantitative real-time PCR (qPCR) in a Thermocycler Mx3000P Stratagene (Massy, France). The reaction mixture contained 5X HOT BIOAmp EvaGreen (Biofidal, Vaulx-en-velin, France) qPCR Mix (no ROX). The 15 μL reactions contained 1 µL of genomic DNA, 0.5 pmol/µL of each forward and reverse primers, 5x EvaGreen qPCR buffer including dNTPs, HOT BIOAmp DNA polymerase, MgCl_2_ at 12.5 mM, EvaGreen dye. The cycling was initiated by one denaturation step at 95 °C for 5 min; followed by 40 cycles of [95 °C for 15 s (denaturation), 64 °C for 20 s (annealing step) and 72 °C for 20 s (elongation) each]. Fluorescence from both the evagreen dyes was recorded at the annealing step. The point at which the PCR product is first detected above a fixed threshold, the thermal cycle threshold (C_t_), was determined for each sample in duplicate, and the average C_t_ of duplicate sample was calculated. The three possible genotypes YY, YC and CC were deduced from the differences between their characteristic cycle threshold values (ΔCt). That is difference of Ct values between the matched and the mismatched primer extension for homozygous rats, and the absence of such a difference in Ct values for heterozygous animals.

### Animal treatment

Experimental research on animals was performed according to an experimental protocol following international guidelines and with approval from the ethics committee of the Veterinary School of Lyon. During vitamin K deficiency experiments, 4 weeks old animals received just after weaning and for 12 weeks a diet deficient in vitamin K_3_ (menadione) without barley (Scientific Animal Food and Engineering, SAFE A04 v231) (designed in this study as diet −K3) *ad libitum*. This diet was composed of corn starch 36.1% (instead of barley rich in vitamin K_1_), wheat 25%, corn 15%, wheat bran 5%, peanut oil 3%, soybean meal 8%, fish hydrolyzate 4% and minerals/vitamins mix 3.9% (without vitamin K_3_). Concentrations of vitamin K_1_ and K_3_ were determined by the supplier and were under the limit of quantification.

In addition, all along the feeding period with the special diet −K3, some animals received menadione bisulfite supplied in the drinking water (200 mg/L of water) which was available *ad libitum*, other animals received daily subcutaneous injections of phylloquinone (10 mg/kg).

Finally, rats were anesthetized with isoflurane and blood was taken by cardiac puncture into citrated heparin lithium tubes. Finally, rats were euthanized with CO_2_ and organs (liver, kidney, lung, heart and testis) of each rat were immediately collected and stored at −20 °C or fixed in a solution of 4% formaldehyde (Microm Microtech, Brignais, France) until analysis.

### Coagulation measurements

After blood collection into citrated tubes, blood samples were centrifuged at 3000 g for 10 minutes and plasmas were used immediately for prothrombin time determinations.

The prothrombin time was determined from 100 µl of fresh citrated plasma and 200 µl of thromboplastin (Neoplastin CI, INR Determination kit, Diagnostica Stago, Asniere, France) using a Biomerieux Option 2 plus (Behnk Electronick, Norderstedt, Germany) according to the manufacturer’s recommendations.

Vitamin K-dependent clotting factor II activity was measured on citrated plasma using a chromogenic method on Konelab 20 (Thermofisher, Cergy Pontoise, France) with Hyphen Biomed reagents. Factor II was activated by incubation of citrate plasma with Echis Carinatus venom (0.25 mg/mL) in Tris-buffer (0, 1 mM Tris Hcl, pH 8.3 at 25 °C, 0.2% BSA) at 37 °C for 10 min. Thus, 50 µg of thrombin chromogenic substrate was added and absorbance was monitored at 405 nm for 1 min.

### Biochemical parameters measurements

After blood collection in heparin lithium tubes, blood samples were centrifuged at 3000 g for 10 minutes and plasmas were frozen at −80 °C for biochemical determinations.

Biochemical parameters were determined from thawed plasma using a chromogenic method on Konelab 20 (Thermofisher, Cergy Pontoise, France) with specific reagents (Termo Fisher Diagnostics, Dardilly, France).

Menaquinone-4 concentrations were determined by HPLC with fluorescence detection as described previously^49^.

### Micro-CT analysis

The μ-CT acquisitions were carried out after euthanasia of the animals by lethal injection of pentobarbital, under isoflurane anesthesia. Three successive periods of acquisition were performed, each period was performed to detect calcifications in specific regions. The first region of interest included lung, aorta and aortic arch regions; the second one, kidneys; the third one, testes. Acquisitions were made with a field of view of 80 mm diameter and 35 mm depth and a pixel size and a layer thickness of 90 µm. A manual segmentation was then performed around each target region (lung/aortic arch/aorta, kidneys and testes). The natural densities of these 4 region of interests being intrinsically different, different thresholds have been set for the segmentation of calcified areas, in order to exclude soft tissue in the quantization. Therefore, different sensitivity was applied for the detection of calcifications for each organ (50 HU for the lungs and kidneys (highest sensing zone, 25 HU for the aortic arch area aorta (intermediate sensing zone) and 30 HU per testis (lowest sensitivity zone).

Volume of calcifications observed in the 4 target regions was then calculated. In some regions of interest, artefacts due to the presence of bone density were detected. These artifacts were not taken into account in the final calculation of calcification volumes.

### Tissue calcium measurements

Tissues were placed in an oven at 50 °C for 72 h then reduced to a powder using a glass mortar. Calcium was extracted from 10 to 50 mg of tissue powder (depending to the organs) in 200 µl to 1 ml of a 10% formic acid solution for at least 2 hours. The supernatant was recovered after centrifugation at 3000 g for 10 minutes and then evaporated at 110 °C. Residues were resuspended with mili-Q water corresponding to half of the volume of the formic acid solution Calcium was assayed using the calcium colorimetric assay kit, (Sigma-Aldrich, l′Isle d’Abeau, Chesnes, France) according to the manufacturer’s recommendations.

### Histology study

Tissues were fixed at least 48 hours in a 4% formaldehyde solution prior to being embedded in paraffin. Von Kossa and hematoxylin & eosin staining were subsequently carried out on 3.5 µm thick sections.

Images of sections were obtained using an Olympus microscope BX50 (Olympus, France) mounted with a Coolpix 4500 camera (Nikon, France). Pictures were treated using eclipsnet software (version 1.20.0).

### Data analysis

Data are presented as the mean ± SD. Statistical analysis was done by using the Tukey’s multiple comparisons test, using GraphPad Prism 6 software (GraphPad, San Diego, CA, USA). *P* < 0.05 was the accepted level of significance. Statistic difference was reported when only a unique variable between groups was modified (either genotype or diet).

## Electronic supplementary material

Supplementary Information
