# Supplementary material for: Missense mutation of VKORC1 leads to medial arterial calcification in rats
Source: Sci Rep. 2018 Sep 13;8:13733. doi: 10.1038/s41598-018-31788-6 (PMC6137107; doi:10.1038/s41598-018-31788-6)
Supplement: Supplementary file 1 — Supplementary Information [file 41598_2018_31788_MOESM1_ESM.pdf]

Missense mutation of *VKORC1* leads to medial arterial calcification in rats

Arnaud Michaux[1], Benjamin Matagrin[1], Jean-Valéry Debaux[1], Leon J. Schurgers[2], Etienne Benoit[1], Virginie Lattard[1,*]

Supplementary information

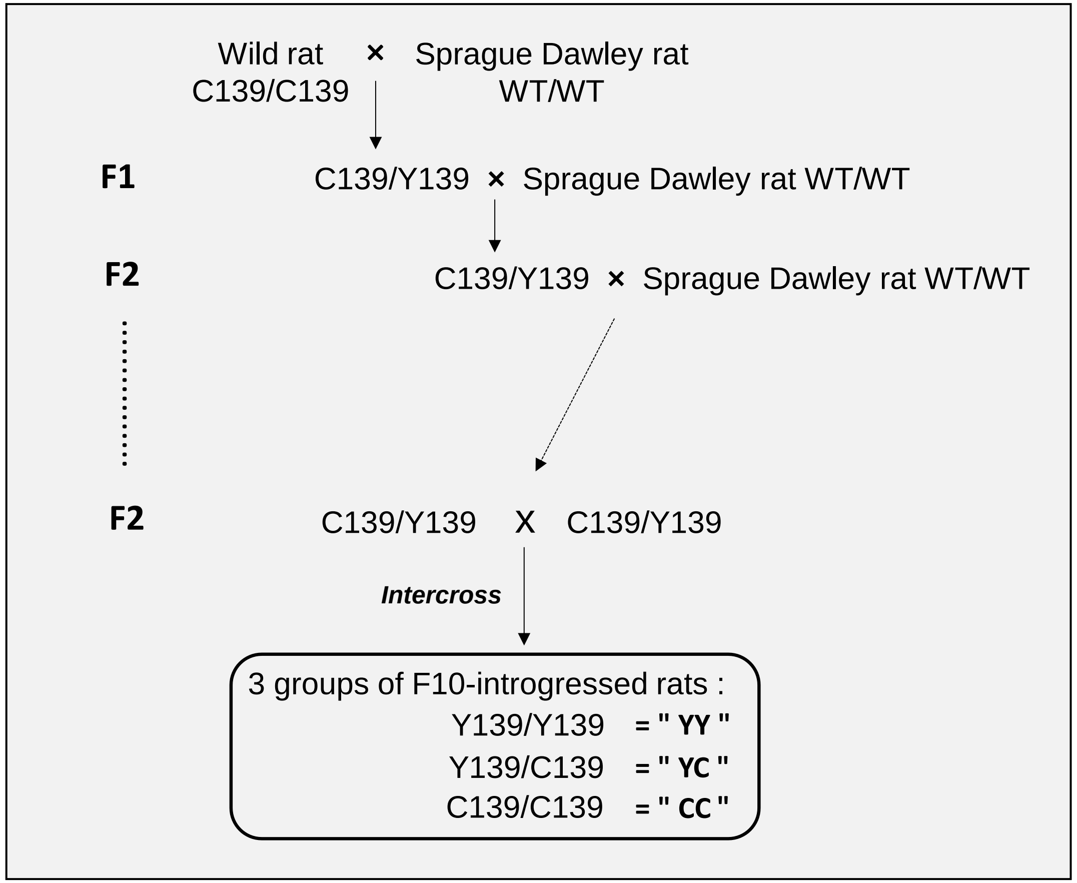

Supplemental figure 1 : Introgression of C139 mutation into Sprague Dawley *Rattus norvegicus* strain

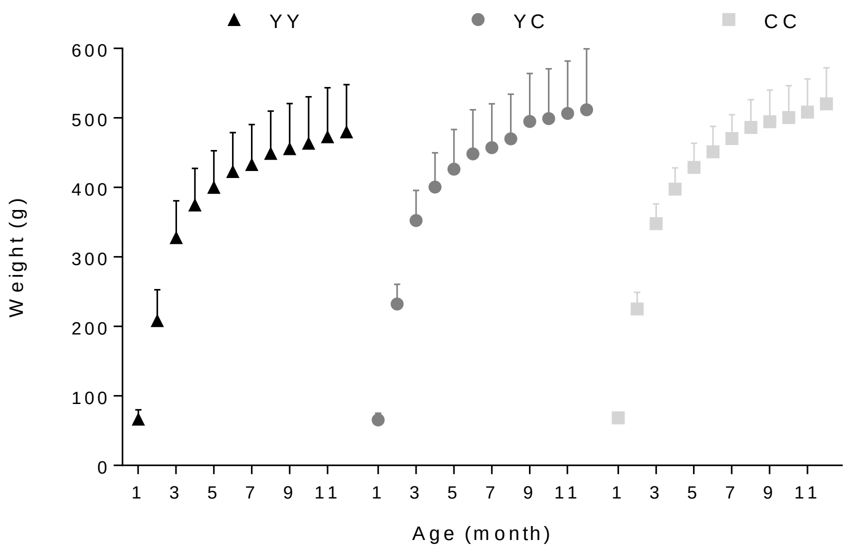

Supplemental Figure 2: Weight charts of YY, YC and CC-F10-introgressed rats fed with a standard diet for 1 year

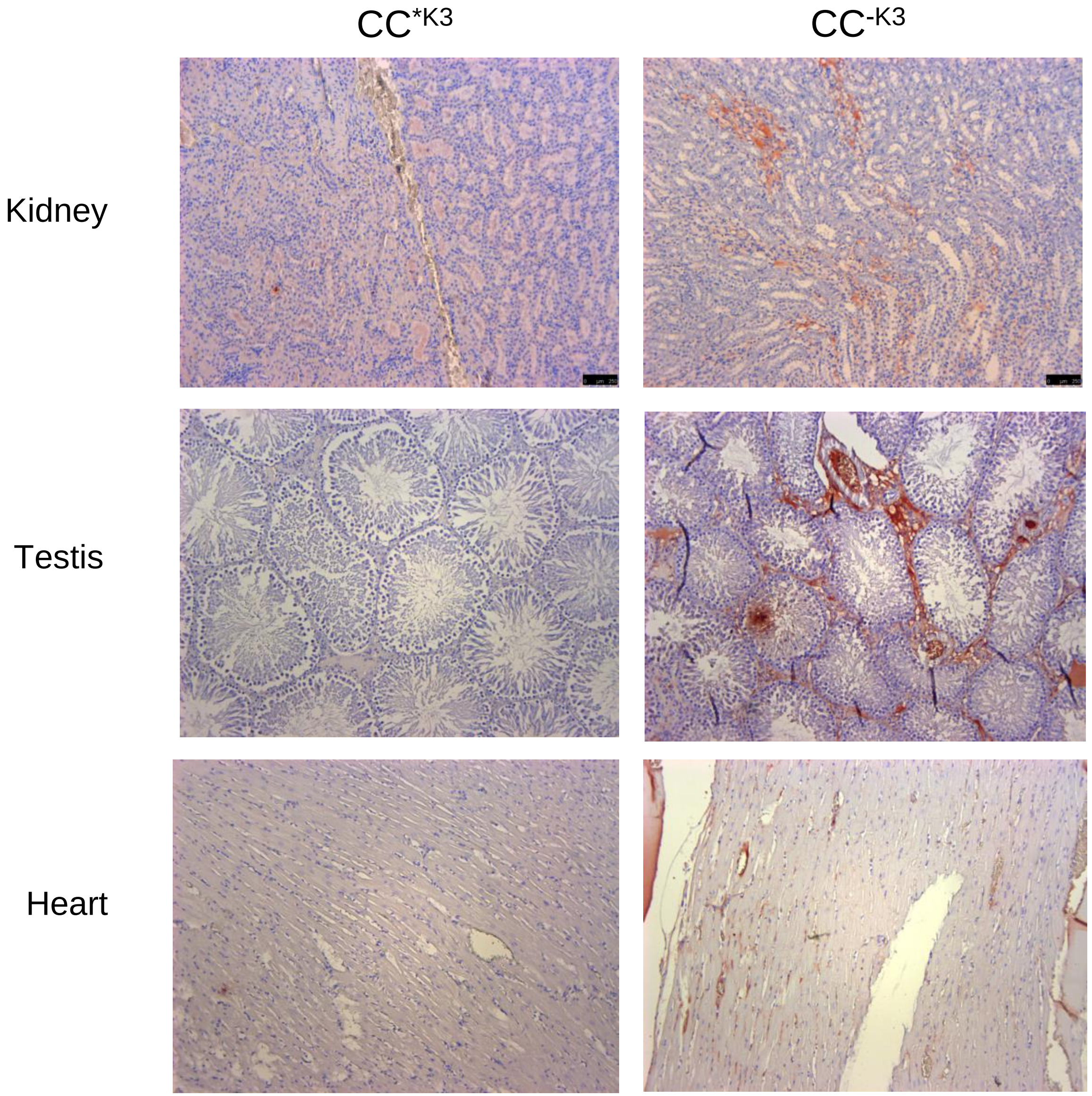

Supplemental figure 3 : ucMGP immunostaining of various tissues of CC rats feed with (CC^{+K3}) or without (CC^{-K3}) vitamin K3 during 12 weeks
